# A Framework to Predict the Molecular Classification and Prognosis of Breast Cancer Patients and Characterize the Landscape of Immune Cell Infiltration

**DOI:** 10.1155/2022/4635806

**Published:** 2022-06-07

**Authors:** Kun Zheng, Zhiyong Luo, Yilu Zhou, Lili Zhang, Yali Wang, Xiuqiong Chen, Shuo Yao, Huihua Xiong, Xianglin Yuan, Yanmei Zou, Yihua Wang, Hua Xiong

**Affiliations:** ^1^Department of Oncology, Tongji Hospital, Tongji Medical College, Huazhong University of Science and Technology, Wuhan 430030, China; ^2^Department of Thyroid and Breast Surgery, Tongji Hospital, Tongji Medical College, Huazhong University of Science and Technology, Wuhan 430030, China; ^3^Biological Sciences, Faculty of Environmental and Life Sciences, University of Southampton, Southampton SO17 1BJ, UK; ^4^Institute for Life Sciences, University of Southampton, Southampton SO17 1BJ, UK

## Abstract

It is known that all current cancer therapies can only benefit a limited proportion of patients; thus, molecular classification and prognosis evaluation are critical for correctly classifying breast cancer patients and selecting the best treatment strategy. These processes usually involve the disclosure of molecular information like mutation, expression, and immune microenvironment of a breast cancer patient, which are not been fully studied until now. Therefore, there is an urgent clinical need to identify potential markers to enhance molecular classification, precision prognosis, and therapy stratification for breast cancer patients. In this study, we explored the gene expression profiles of 1,721 breast cancer patients through CIBERSORT and ESTIMATE algorithms; then, we obtained a comprehensive intratumoral immune landscape. The immune cell infiltration (ICI) patterns of breast cancer were classified into 3 separate subtypes according to the infiltration levels of 22 immune cells. The differentially expressed genes between these subtypes were further identified, and ICI scores were calculated to assess the immune landscape of BRCA patients. Importantly, we demonstrated that ICI scores correlate with patients' survival, tumor mutation burden, neoantigens, and sensitivity to specific drugs. Based on these ICI scores, we were able to predict the prognosis of patients and their response to immunotherapy. Together, these findings provide a realistic scenario to stratify breast cancer patients for precision medicine.

## 1. Introduction

Breast cancer (BRCA) has now risen to become the most common malignant tumor throughout the world and the second leading cause of cancer-related death in women. The US added 270,000 new diagnosed cases and more than 40,000 deaths in 2020 [1, 2]. Due to its considerable influence on public health worldwide, the molecular mechanisms of breast cancer-like associated genes and pathways [3], metastasis [4], and drug responses [5, 6] have been widely studied. Over recent years, there have been great advances in treatment strategies for BRCA including surgical resection, chemotherapy, radiotherapy, targeted therapy, and endocrine therapy. However, due to factors such as local recurrence, distant metastasis, and high tumor heterogeneity, the prognosis of BRCA patients is still unsatisfactory [7, 8].

The tumor microenvironment (TME) includes tumor cells, tumor-infiltrating lymphocytes (TILs), and stromal components, which can serve as a key mediator of cancer progression and treatment outcome [9, 10]. Over the past few years, numerous studies have shown that TILs play key roles in tumor extension, recurrence, metastasis, and therapeutic response to cancer immunotherapy [11–13]. For example, naive CD8^+^ T cells, when bound and activated by antigen-presenting dendritic cells, would become effector T cells, which could then recognize and kill tumor cells by releasing granzymes to induce apoptosis [14]. Chemokines secreted by tumor cells, such as C-C Motif Chemokine Ligand 2, C-C Motif Chemokine Ligand 5, and colony stimulating factor 1, can recruit M2-type tumor-associated macrophages, and their abundance in TME correlates with a poor prognosis [15].

Cancer immunotherapy, including immune checkpoint inhibitors, has provided clinical benefit to the treatment of many BRCA patients through direct or indirect effects on TILs, reversing the TMEs to immune-permitted environments from immunosuppressive ones [16]. Promising outcomes in response to antibodies to programmed cell death 1 (PD-1) or antibodies to programmed cell death ligand 1(PD-L1) therapy for BRCA have been reported in recent years [17–19]. However, the immune microenvironment of BRCA remains poorly understood, and this treatment can only benefit a limited proportion of patients [20, 21]. Therefore, identification of potential biomarkers is in urgent clinical need to enhance precision prognosis and therapy stratification for BRCA patients.

In our study, the gene expression profiles of 1,721 BRCA patients were analyzed by CIBERSORT and ESTIMATE algorithm, by which we obtained a comprehensive intratumoral immune landscape. The immune cell infiltration (ICI) patterns of BRCA were classified into 3 separate subtypes according to the infiltration levels of 22 immune cells. The differentially expressed genes (DEGs) between these subtypes were further identified, and ICI scores were calculated to assess the immune landscape of BRCA patients. Importantly, we demonstrated that ICI scores correlate with patients' survival, tumor mutation burden (TMB), neoantigens, and sensitivity to specific drugs. Based on these ICI scores, we were able to predict the prognosis of patients and their response to immunotherapy. Together, these findings provide a realistic scenario to stratify BRCA patients for precision medicine.

## 2. Materials and Methods

### 2.1. Source of Cohort Datasets and Immune-Related Data and Preprocessing

The training datasets of BRCA for this study were integrated from two separate cohorts (TCGA-BRCA and Yau-cohort), with only tumor samples retained. The expression profile data of TCGA-BRCA cohort (considering only protein-coding mRNA) were downloaded from The Cancer Genome Atlas (TCGA) database by Genomic Data tools (https://portal.gdc.cancer.gov/projects/TCGA-BRCA). The fragments per kilobase million values were downloaded via TCGAbiolinks [22] package and transformed to transcripts per million, with the ensemble ID matrix converted to a gene symbol matrix and other forms for subsequent analysis. The Yau-cohort dataset [23], integrated by Dr. Yau from four studies (GSE2034, GSE5327, GSE7390, and NKI295), was downloaded from the online database University of California Santa Cruz (UCSC) Xena browser (https://xenabrowser.net/). It contains the gene expression matrix along with clinical information of 682 breast cancer patients. At last, the batch effects caused by nonbiological technical bias were reduced through “Combat” algorithm [24].

The clinical information for the TCGA-BRCA cohort was extracted from the pan-cancer data, which included age, sex, clinicopathological stage, TNM stage, and PAM50 subtype, and only overall survival (OS) was considered. The Yau-cohort cohort considered OS, age, and PAM50 subtype. A total of 1721 breast cancer samples were generated after kicking out the samples with incomplete clinical information and survival time and male breast cancer samples. To analyze the efficiency of immunotherapy, the R package IMvigor210CoreBiologies [25] obtained from the work of Snyder et al. was used as a validation dataset, which included expression profiles, survival outcomes, and immunotherapy response results in metastatic uroepithelial cancer patients treated with anti-PD-L1 agent atezolizumab.

### 2.2. Consensus Clustering of TME Immune Cell Infiltration

The CIBERSORT and ESTIMATE algorithms were combined to reckon the abundance and infiltration levels of 22 immune cell species of the integrated BRCA cohort [26, 27]. LM22 signature matrix, which provided a gene expression signature set of 22 immune cell subtypes and CIBERSORT source code, was downloaded from the CIBERSORT website (https://cibersortx.stanford.edu/). Unsupervised clustering analysis of ICI of each sample was performed using R package “ConsensusClusterPlus” [28], which classified the tumor ICI pattern of BRCA patients into different subtypes (maxK, the maximum number of classifications *K* = 3). 90% of the samples have been repeated 500 times, ensuring stability of the classification. Calculation of distances was measured using Spearman's distance measure and Ward's linkage.

### 2.3. Identification of DEGs between ICI Subtypes and Gene Signature Generation

To identify genes associated with ICI patterns, we applied the R package “limma” [29] to determine the DEGs between different ICI subtypes and plotted the DEGs heatmap using the “ComplexHeatmap” R package [30]. The significance cutoff criteria used to distinguish DEGs were set as fold change (FC) > 1.5 and adjusted false discovery rate (FDR) < 0.05.

To quantify the ICI pattern of a single tumor patient, we established a scoring system, ICI gene signature, to confirm the ICI pattern for each BRCA patient, and we termed it ICIscore. The steps to establish an ICI gene signature are as follows: Firstly, the DEGs were analyzed by unsupervised cluster analysis using the R package “ConsensusClusterPlus.” The maximum number of classifications is 3, and the distances were calculated using Pearson's distance measure and complete linkage, which divided the TCGA-BRCA cohort into 3 genomic clusters, namely, ICI gene clusters A, B, and C. And then, Pearson's correlation analysis was done on the mRNA expression values of all TCGA samples with the three gene clusters, and the DEGs with positive and negative correlation with clustering features were, respectively, defined as ICI signature genes A and B. Then, the Boruta algorithm was used to reduce the dimensionality of different ICI signature genes. Finally, two total scores were calculated using single sample gene set enrichment analysis (ssGSEA): (1) ICI score A which is from ICI signature gene A and (2) ICI score B which is from ICI signature gene B: ICI score = ICI score A − ICI scoreB, with median as the cutoff value to determine the high ICI group and the low ICI group. When survival analyses were performed with ICI score groups, we only picked out genes whose *P* value < 0.05 in the univariate survival analysis. Principal component analysis (PCA) was used to calculate the ICI score for each patient, and PCA1 was calculated as the signature score using PCA: ICI score = |PCA1 positive| + |PCA1 negative|. Patients were reclassified as high and low ICI score groups using the median as the cutoff value.

### 2.4. Collection and Analysis of Somatic Mutation Data

The copy number variant (CNV) data of TCGA-BRCA cohort were obtained from the firehose database (http://gdac.broadinstitute.org/), and mutant annotation format (MAF) files were downloaded from the cBioportal database (http://www.cbioportal.org/). To determine the TMB of BRCA, we matched TCGA-BRCA MAF files with ICI-related expression profiles and used the R package “maftools” to calculate the TMB [31]. Based on the OncodriveCLUST algorithm [32], we used the positional information of the somatic mutation sites to cluster the driver genes from different ICI score groups and used the “maftools” package to draw a waterfall map of the top 25 driver genes in the two groups. The CNV analysis was performed with the GenePattern online analysis tool (https://www.genepattern.org/) and visualized with the “maftools” package.

### 2.5. Identification of Sensitive Drugs and Other Biological Processes Correlated with ICI Gene Signatures

The drug.txt is a dataset for the sensitivity and response of cancer cells to therapeutic drugs obtained from the online database Genomics of Drug Sensitivity in Cancer (GDSC), used to predict IC50 with R package “pRRophetic” [33, 34]. We assessed the IC50 values in both ICI score groups using Wilcoxon's test, then compared the differences in sensitivity between ICI score groups on more than 100 drugs, and graphed the top 12 (according to *P* value) differentially response drugs. Wilcoxon's test was also used to compare the differential expression of neoantigen between ICI score groups. Sample data used for predicting neoantigen number in the TCGA-BRCA cohort were from a research already published in 2015 by Rooney et al. [35].

Additionally, we separately performed Gene Ontology (GO) enrichment analysis of ICI gene signatures A and B via the “org.Hs.eg.db” R package to explore the biological process, cellular composition, and molecular function that they may participate in. After differential expression analysis with the “limma” package for high or low ICI score groups, the differentially expressed genes were subjected to Gene Set Enrichment Analysis (GESA), and the gene sets “h.all.v7.2.symbol” were downloaded from the Molecular Signatures Database (MSigDB) (http://www.gsea-msigdb.org/gsea/msigdb) for running GESA analysis. To confirm the difference in the efficacy of anti-PD-L1 immunotherapy between the two ICI score groups in the validation cohort, IMvigor210, objective remission rate bar graphs were plotted for the ICI score groups using “GSVA” R package [36].

### 2.6. Statistical Analysis

All statistical analyses were performed using R software (version 3.6.2). Wilcoxon's test was used to compare the differences between two groups, and the Kruskal-Wallis test was used to compare the differences between more than two groups. The Kaplan-Meier survival curves were plotted using the R package “survminer” for different subgroups, such as ICI clusters, ICI gene clusters, ICI gene signatures, and TMB subgroups, in relation to survival. Log-rank test was used for statistically significant differences. The R packages “ComplexHeatmap” and “ggplot” were used to draw heatmaps, scatter plots, violin plots, and other plots. Correlation coefficients were calculated by using Spearman's analysis. Two-tailed *P* < 0.05 was considered a statistically significant difference.

## 3. Results

### 3.1. The Immune Cell Infiltration (ICI) Landscape in BRCA Immune Microenvironment

**Figure 1:**
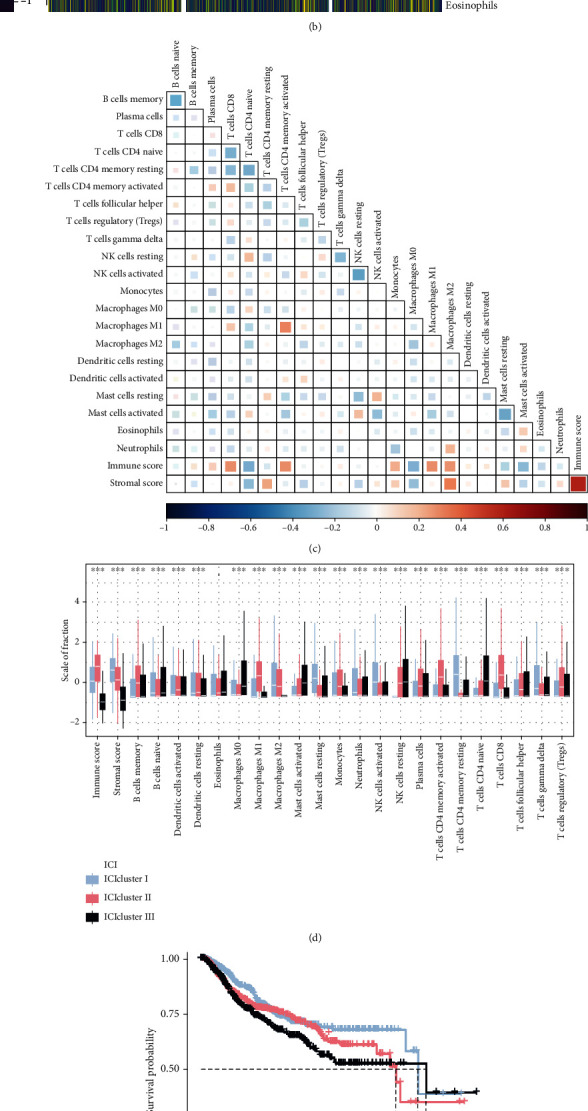
The immune-cell infiltration (ICI) landscape in BRCA immune microenvironment. (a) PCA of integration of expression profiles of TCGA-BRCA and Yau cohorts by Combat algorism to eliminate batch effects of different cohorts. (b) Heatmap with unsupervised clustering analysis of tumor-infiltrating immune cells in TCGA-BRCA and Yau cohorts. (c) Hotspot plot for correlation matrix of immune cells in three ICI clusters, including their immune scores and stromal scores. Red indicates positive correlation, and blue indicates negative correlation. (d) Box plot for abundance of each immune infiltrating cells in the three ICI clusters. The asterisks represent the statistical *P* value (Kruskal-Wallis test, ^∗^*P* < 0.05,  ^∗∗^*P* < 0.01, and^∗∗∗^*P* < 0.001). (e) Survival analysis for three ICI clusters of 1721 breast cancer patients from TCGA-BRCA and Yau cohorts using Kaplan-Meier curves. The log-rank test showed that *P* = 0.007. Violin plots of the differential expression of (f) PD1 and (g) PD-L1 (only for TCGA-BRCA cohort) among the three ICI clusters. The statistical differences among ICI clusters were compared by the Kruskal-Wallis test (*P* < 0.001).

We first performed PCA of integrated gene expression profiles of 1,721 BRCA patients from the training cohort consisting of the TCGA-BRCA and Yau cohorts by using Combat algorithm to eliminate batch effects across cohorts (Figure 1(a)). Subsequently, we performed the CIBERSORT algorithm combined with the ESTIMATE algorithm to determine the abundances of 22 immune cells as well as the enrichment scores of stromal cells (stromal score) and immune cells (immune score) in BRCA patients in this cohort (Supplementary Table 1). We performed an unsupervised cluster analysis of this cohort by ConsensusClusterPlus R package to divide BRCA patients into 3 separate subtypes based on ICI patterns, referred to as ICI clusters I, II, and III, respectively (Figure 1(b)). A hotspot matrix of correlation coefficients was created to demonstrate the overall landscape of interactions among immune cells in the TME of BRCA patients, including their immune scores and stromal scores (Figure 1(c)).

To explore the inherent biological differences between the different ICI subtypes, we compared the composition of immune cells in the 3 ICI clusters. As shown in Figure 1(d), ICI cluster I was characterized by high level M2 macrophages, neutrophils, resting mast cells, activated natural killer (NK) cells, resting CD4^+^ T cells, and gamma delta T cell infiltration; patients from ICI cluster II had a higher density of memory B cells, activated dendritic cells, resting dendritic cells, M1 macrophages, monocytes, memory activated CD4^+^ T cells, CD8^+^ T cell, follicular helper T cells, plasma cells, and regulatory T cells, while ICI cluster III displayed an increase in naïve B cells, naïve CD4^+^ T cells, resting NK cells, M0 macrophages, and activated mast cell infiltration. Survival analysis conducted on these 3 ICI subtypes showed significant differences among them, with ICI clusters I and II being associated with better prognosis and patients in ICI cluster III having a poorer OS (log-rank test, *P* = 0.007; Figure 1(e)). In addition, we analyzed the expression of PD-1 and PD-L1 in each ICI subtype (Figures 1(f) and 1(g)). The results of Kruskal-Wallis test showed higher expression of PD-1 and PD-L1 in ICI cluster II, while their expressions were lowest in ICI cluster III.

### 3.2. Identification and Comprehensive Analysis of Immunogenic Gene Clusters

**Figure 2:**
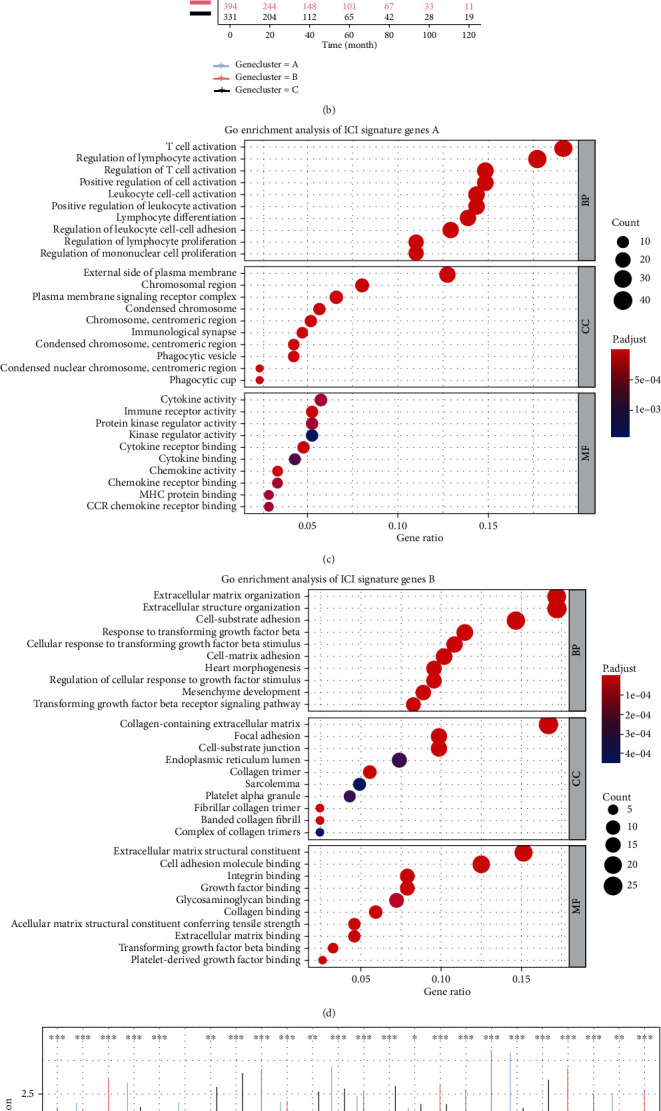
Identification and comprehensive analysis of immunogenic gene clusters. (a) Heatmap with unsupervised clustering analysis of all DEGs in the three ICI patterns, dividing TCGA-BRCA patients into three genomic clusters, defined as ICI gene clusters A-C. Rows represent genes and columns represent samples. (b) Survival analysis for the three ICI gene clusters in TCGA-BRCA patients using Kaplan-Meier curves. The log-rank test showed that *P* = 0.04. Functional annotation of ICI gene clusters (c) A and (d) B using GO enrichment analysis. The circle size of the bubble plots represented the number of enriched genes. (e) Box plot for abundance of each immune infiltrating cell in the three ICI gene clusters. The asterisks represented the statistical *P* value (Kruskal-Wallis test, ^∗^*P* < 0.05,  ^∗∗^*P* < 0.01, and‑*P* < 0.001). Violin plots of the differential expression of (f) PD1 and (g) PD-L1 among the three ICI gene clusters. The statistical differences among ICI gene clusters were compared by Kruskal-Wallis test (*P* < 0.001).

To elucidate the potential characteristics of the different immunophenotypes, we conducted the limma package to identify DEGs among ICI clusters I, II, and III (FC = 1.5, FDR = 0.05). Based on the above cutoffs, we identified 665 DEGs (213 in ICI cluster I, 239 in ICI cluster II, and 213 in ICI cluster III; Supplementary Table 2) and used the ComplexHeatmap package to generate a heatmap of all DEGs. Hereafter, we focused our analysis on the TCGA-BACR cohort as it had comprehensive information on clinical aspects. We performed an unsupervised clustering analysis of these DEGs and divided the TCGA-BRCA cohort into 3 distinct ICI genomic phenotypes, named ICI gene clusters A, B, and C, respectively (Figure 2(a)). We defined all above DEGs with positive association with these 3 ICI gene clusters as ICI signature genes A, while the rest of DEGs were termed as ICI signature gene B. By down-dimensioning the ICI signature genes using Boruta algorithm to reduce redundant genes, we finally obtained 216 genes in ICI signature gene A and 164 in ICI signature gene B (Supplementary Table 3).

In Figure 2(b), we figured out the prognostic differences among these ICI gene clusters, and we confirmed that ICI gene clusters A and B had a better prognosis, and the prognosis of ICI gene cluster C was poorer (log-rank test, *P* = 0.04). Figures 2(c) and 2(d) show the results of gene ontology (GO) enrichment analysis of both ICI signature gene groups in the 3 functional groups, biological process, cellular component, and molecular function, respectively, which were significantly enriched in items related to immunity. Given that the immune system can exert both antitumor and protumor activities [37, 38], we next explored the level of immune infiltration cells among different gene clusters, and the box plot showed that gene clusters A and B with favorable prognosis had higher immune and stromal scores (Figure 2(e)). Besides, there were the highest infiltrations of M1 macrophages, CD8^+^ T cells, memory activated CD4^+^ T cells, memory B cells, activated dendritic cells, and plasma cells within ICI gene cluster B, showing the active immune phenotype. In contrast, the level of infiltration of these TILs was very low in the poorly prognosed ICI gene cluster C. The three ICI gene clusters also showed significant differences in the expression levels of PD-1 and PD-L1. There were relatively high expression levels of PD-1 and PD-L1 in ICI gene clusters A and B, while they had the lowest expression levels in ICI gene cluster C (Figures 2(f) and 2(g)). From the above comprehensive analysis of immunogenic gene clusters, we demonstrated that there is a significant correlation between the level of ICI and prognosis in different gene clusters.

### 3.3. Immune-Cell Infiltration (ICI) Score Construction

**Figure 3:**
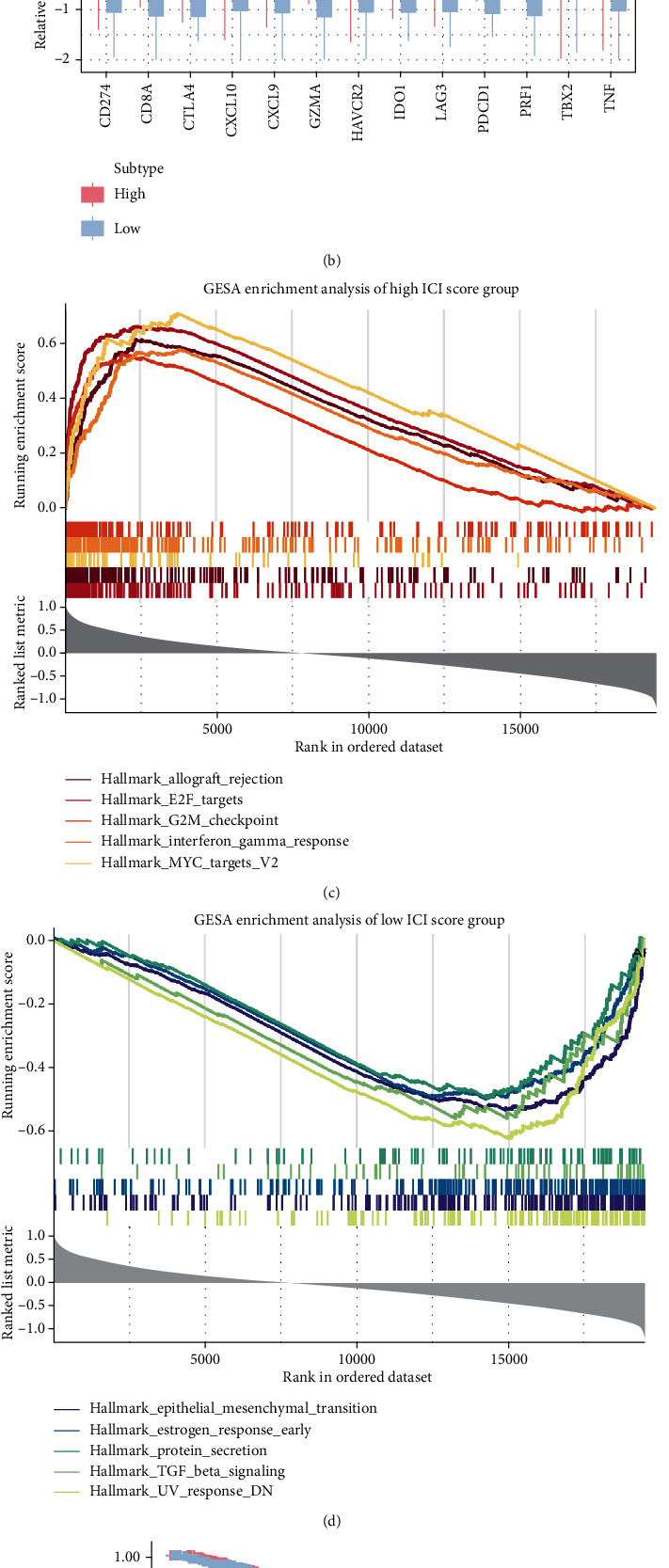
Immune-cell infiltration (ICI) score construction. (a) Alluvial diagram showing the distribution of ICI gene clusters in different ICI score groups and survival status of TCGA-BRCA patients. (b) Box plot for the relative expression of immune checkpoint-associated genes in different ICI score groups. Among them, *CD274*, *HAVCR2*, *CTLA4*, *LAG3*, *PDCD1*, and *IDO1* are inhibitory genes, and *CD8A*, *GZMA*, *PRF1*, *CXCL10*, *CXCL9*, *TNF*, and *TBX2* are stimulatory genes. The asterisks represent the statistical P-value (Kruskal-Wallis test, ^∗^*P* < 0.05,  ^∗∗^*P* < 0.01, and^∗∗∗^*P* < 0.001). GESA enrichment maps for (c) high and (d) low ICI score groups. Allograft rejection, E2F targets, G2M checkpoint, interferon gamma response, and MYC target V2 pathways were enriched in the high ICI score group. Epithelial mesenchymal transition, estrogen response early, protein secretion, TGF-*β* signaling, and UV response pathways were enriched in the low ICI score group. (e) Survival analysis for high or low ICI score groups (calculated by using PCA) in TCGA-BRCA patients using Kaplan-Meier curves. The log-rank test showed that *P* = 0.033.

Given the individual heterogeneity of the TME, we quantified the ICI pattern of BRCA patients. We calculated 2 summary scores, that is, ICI score A from ICI signature gene A and ICI score B from ICI signature gene B, using ssGSEA. The ICI score of each patient of TCGA-BRCA cohort was determined using the difference between ICI scores A and B. The high ICI score group and low ICI score group were defined using median as the cutoff value. The distribution of ICI scores and survival of patients in ICI gene clusters are shown in Figure 3(a) and Supplementary Table 4.

We further analyzed the differences in the expression of immunoreactive-related genes in the high or low ICI score groups to determine the status of immune activity or tolerance in each group. Among them, *CD274*, *HAVCR2*, *CTLA4*, *LAG3*, *PDCD1*, and *IDO1* were chosen as immune inhibitory genes [39], while *CD8A*, *GZMA*, *PRF1*, *CXCL10*, *CXCL9*, *TNF*, and *TBX2* as immune stimulatory genes [40]. As we can observe in Figure 3(b), the expression levels of all immunoreactive-related genes were significantly elevated in the high ICI score group. We performed the differential expression analysis of genes in the high or low ICI score groups using the limma package (FC = 1.5, FDR = 0.05) and obtained 890 DEGs. Our subsequent GSEA analysis of these DEGs showed that the high ICI score group was significantly enriched in allograft rejection, E2F targets, G2M checkpoint, interferon gamma response, and MYC target V2 pathways, while the low ICI score group was mainly enriched in epithelial mesenchymal transition, estrogen response early, protein secretion, TGF-*β* signaling, and UV response pathways (Figures 3(c) and 3(d) and Supplementary Table 5). In addition, when we compared the relationship between ICI scores and prognosis, we only selected genes with *P* value < 0.05 in the univariate survival analysis. We then used PCA to calculate the ICI score for each patient. Patients were redivided into high and low ICI score groups using the median value as the cutoff. The Kaplan-Meier curves in Figure 3(e) indicated that patients of the high ICI score group have significantly longer survival than those of the low ICI score group (log-rank test, *P* = 0.033).

### 3.4. Correlation between Immune Cell Infiltration (ICI) Scores and Tumor Mutation Burden (TMB)

**Figure 4:**
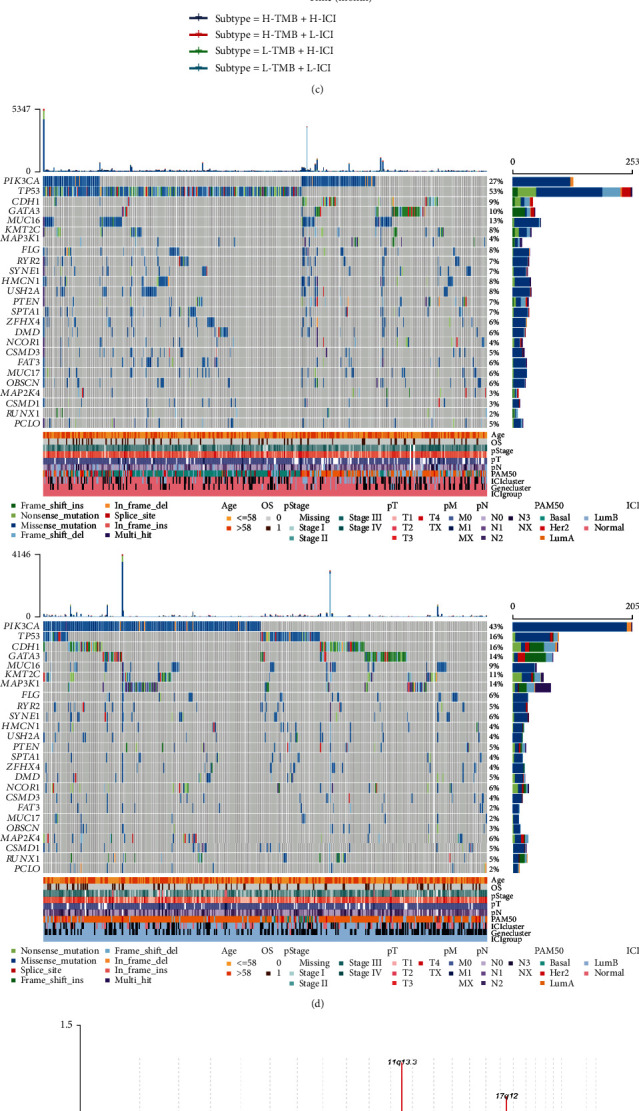
Correlations between immune-cell infiltration (ICI) scores and tumor mutation burden (TMB). (a) Differences in TMB between high or low ICI score groups (Kruskal test, *P* < 0.001). (b) Scatter plot of correlation between ICI scores and the mutational burden in the TCGA-BRCA cohort (Pearson's correlation =0.133, *P* < 0.001). (c) Stratified survival analysis of TCGA-BRCA patients stratified by both TMB and ICI scores (calculated by using PCA) using Kaplan Meier curves. The log-rank test showed that *P* = 0.039. (d) Waterfall plots of the top 25 significantly driver mutated genes in the high (left) or low (right) ICI score groups. Each column represents for individual patients, and the bar plot on top shows the TMB. GISTIC2.0-based copy number variant (CNV) analysis of (e) high or (f) low ICI score groups visualized by maftools.

Numerous studies have suggested that the immune phenotype may be associated with alterations in the tumor genome [41, 42]. To validate this hypothesis, we tested the relationship between TMB and ICI scores in the TCGA-BRCA cohort and found that patients in the high ICI score group had more TMB (Kruskal test, *P* = 0.002; Figure 4(a)). Besides, the scatter plot of the association between TMB and ICI scores also showed a positive association (Pearson′s correlation = 0.324, *P* < 0.001; Figure 4(b)). In our stratified survival analysis, which divided patients into different subgroups according to TMB and ICI scores (calculated by using PCA), we found that patients with high level of TMB and low ICI scores had the worst prognosis (log-rank test, *P* = 0.039; Figure 4(c)). We also performed clustering analysis by using the position information of somatic mutations to identify mutation driver genes in different ICI score subgroups, and mapped the waterfall of the top 25 most significant mutation driver genes using the maftools package (Figure 4(d)). Expression profiles of patients in distinct ICI score groups in the TCGA-BRCA cohort were matched with CNV data downloaded from the firehose database, and the GISTIC2.0 module of the GenePattern online tool was used to analyze the status of CNV in different groups. The results from the analysis were visualized using the maftools package and are presented in Figures 4(e) (high ICI score group) and Figure 4(f) (low ICI score group). We found that both high and low ICI score groups had many copy number variations, but high ICI score groups had more CNVs. The regions significantly amplified in the high ICI score group of patients involved 11q13.3, 17q12, and 8q24.21, while 11q13.3 spanned the *CCND1* gene. Significantly deleted regions in the high ICI score group included 9p21.3, which spans the tumor suppressor genes *CDKN2A* and *CDKN2B*.

### 3.5. Integrative Analysis of Immune Cell Infiltration (ICI) Scores on Drug Response

**Figure 5:**
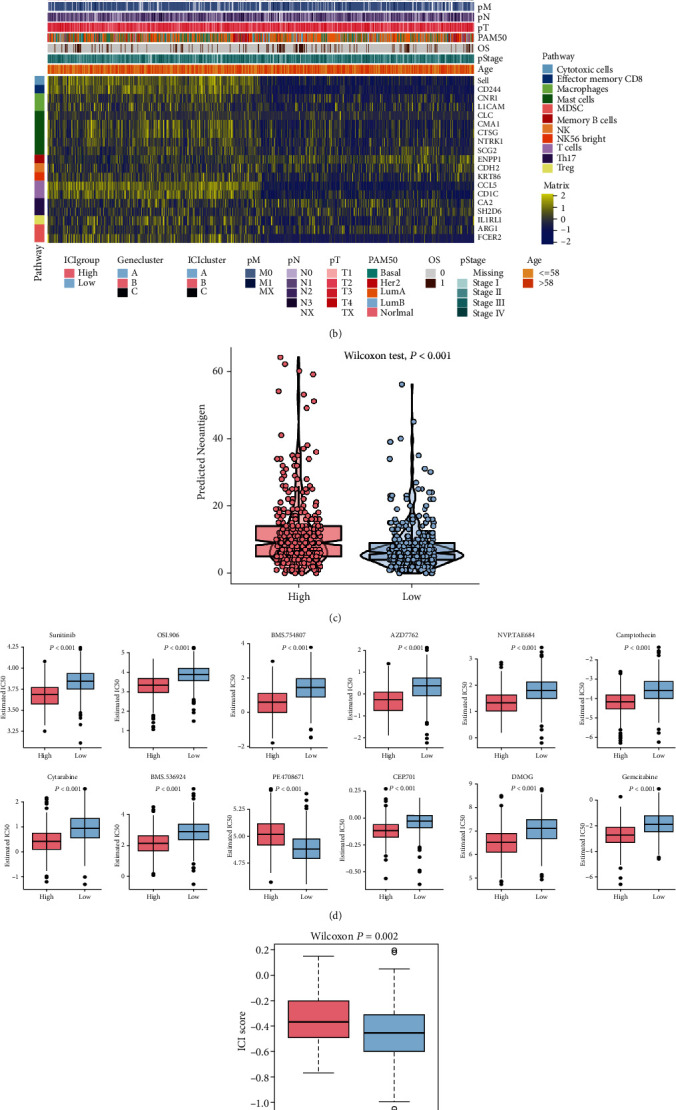
Integrative analysis of immune-cell infiltration (ICI) scores on drug response. Heatmap of immune-related genes in different ICI score groups. The immune-related genes were divided into two groups: (a) gene set 1 and (b) gene set 2. The ICI cluster, ICI gene cluster, PAM50 subtype, age, tumor stage, and survival status were used as patient annotations. (c) Differences in neoantigen between high or low ICI score groups. (d) Comparison of drug sensitivity in high or low ICI score groups (Wilcoxon's test, *P* < 0.001). The box plots show the differences in IC50 values for the top 12 drugs sorted by *P* value. (e) Boxplot for the distribution of ICI scores of patients in different anti-PD-L1 therapeutic responses in IMvigor210 cohort (Wilcoxon's test, *P* = 0.002). (f) Survival analysis for high or low ICI score groups in IMvigor210 cohort patients using Kaplan-Meier curves. The log-rank test showed that *P* = 0.02. (g) Bar graph showing the proportion of patients with various clinical responses (responder: complete response (CR)/partial response (PR); nonresponder: stable disease (SD)/progressive disease (PD)) to anti-PD-L1 immunotherapy in the high or low ICI score groups of the IMvigor210 cohort.

Furthermore, we selected genes from various pathways related to tumor immune processes and classified the immune-related genes of our interest into gene set 1 (Figure 5(a)) and gene set 2 (Figure 5(b)) and then created a heatmap of these genes in the high or low ICI score groups. From Figure 5(a), we found that most of the genes related to diverse immune pathways were upregulated in the high ICI score group and downregulated in the low ICI score group. Figure 5(b) shows a significant increase of genes related to pathways such as cytotoxic cells, effector memory CD8, macrophages, and T cells in the high ICI score group. Both Figures 5(a) and 5(b) show that patients with Luminal A BRCA are mostly enriched in the low ICI score group. In addition to TMB, we also noted the positive correlation between ICI scores and neoantigens (Wilcoxon's test, *P* < 0.001) (Figure 5(c)). We downloaded a dataset of drugs sensitive to the treatment of cancer from the GDSC website, from which we compared the differences in the sensitivity of high or low ICI groups to more than 100 drugs used to treat tumors (Supplementary Table 6). The top 12 drugs with differential treatment responses according to *P* value ranking are illustrated in Figure 5(d), from which it was clear that high ICI scores may lead to increased sensitivity of BRCA to drugs such as imatinib, CCT007093, MK-2206, CHIR-99021, FH535, and KIN001-135. All the above results may provide new perspectives for investigating the role of individual gene mutations in the immune microenvironment and immunotherapy of cancer.

In recent years, blockade therapy targeting immune checkpoints has emerged as a mainstream immunotherapy with the potential to significantly improve the survival of cancer patients, but only small numbers of patients have responded to this treatment [17, 18]. Markers that can effectively predict the effect of immunotherapy are limited; therefore, to validate the role of ICI scores constructed in BRCA patients in predicting patients' response to immunotherapy, we selected the IMvigor210 cohort of metastatic uroepithelial cancer patients with immunotherapy received as a validation cohort to test the potential to forecast immunotherapy benefit of the ICI scores we established. Encouragingly, in the IMvigor210 cohort, we found that ICI scores were in a significantly positive correlation with the objective response rate (ORR) for anti-PD-L1 therapy (Wilcoxon's test, *P* = 0.002; Figure 5(e)). Moreover, in this cohort, patients with high ICI scores had significantly longer survival (log-rank test, *P* = 0.02; Figure 5(f)). What is more, we found the high ICI score group had a higher ORR after anti-PD-L1 treatment (Figure 5(g)). In conclusion, these data suggest that the ICI scores can predict the responses to immunotherapy.

## 4. Discussion

Immunotherapy has changed the treatment and prognosis for many malignancies. In recent years, immunotherapy using checkpoint blockades have proven to generate unprecedented and durable responses in patients suffering from diverse cancers [43–45]. In BRCA, building on the favorable results of the Phase III IMpassion130 trial [46] and the Phase III KEYNOTE-355 trial [47], the U.S. FDA has accelerated approval for the PD-L1 inhibitor atezolizumab as well as the PD-1 inhibitor pembrolizumab, combined with chemotherapy, for the treatment of locally advanced or metastatic PD-L1-positive triple-negative BRCA (TNBC) patients in 2019 and 2020, respectively [48]. In recent years, there has been increasing evidence that patterns of the immune system play a key role in determining both the response to treatment and survival of BRCA patients [49]. These data and the clinical use of immune checkpoint blockers in a variety of solid tumors have also demonstrated striking success [50, 51]. Stromal TIL concentration shows a linear relationship with clinical outcome in different clinical subtypes of BRCA [49]. For example, it has been shown that HER-2+ BRCA and TNBC have higher levels of TILs and PD-L1 expression in TME at diagnosis than luminal BRCA, which can be predicted to benefit more from adjuvant and neoadjuvant chemotherapy, are more likely to respond to PD-1/PD-L1 blockade, and have longer survival [52–54]. However, the use of immunotherapy in BRCA remains limited and only a minority of patients would benefit from it. Poor immunogenicity, T-cell infiltration in TME, and enhanced immunosuppression have been identified as potential challenges to successful immunotherapy for BRCA [55]. Therefore, the development of more efficient biomarkers for predicting response and resistance to therapy, as well as the recognition of environmental modifiers to immunity (mutational load, neoantigens, and sensitive combination therapeutics), is important to improve the efficacy of immunotherapy. It will be of great help to choose the appropriate timing and patients for immunotherapy, patients should be detected markers of immunotherapy response when initial diagnosis, and immunotherapy should be used in treatment as early as possible [56]. In this study, we developed a method to quantify ICI in TME of BRCA patients—ICI score, and our results demonstrated that this score can be used as a predictor to assess the effectiveness and prognosis of immunotherapy.

Many studies have demonstrated the importance of an abundant and active BRCA TME in forecasting the response of tumor patients to immunotherapy [57, 58]. For example, tumors with increased TILs, positive PD-L1, and elevated tumor-infiltrating CD8^+^ T cells exhibit a higher response rate to immunotherapy. Such tumors are considered “inflamed” or “hot” tumors. In contrast, “noninflamed” or “cold” tumors with lower TILs, PD-L1 expression, and CD8^+^ T cell infiltration are less likely to respond to immunotherapy [56]. In this study, we analyzed the ICI patterns of 1721 BRCA samples from the integrated cohort and classified BRCA into three separate immune subtypes and ICI clusters I, II, and III. The results of our analysis suggested that patients in ICI clusters I and II with higher TILs infiltration, PD-L1 expression, and high immune scores had longer survival. This is consistent with previous studies [35, 59]. These findings illustrated that the preexistent immune responses in TME can have an impact on the prognosis of BRCA patients as well as on the degree of benefit from immunotherapy. However, it is not sufficient to rely solely on the immune phenotype of the tumor to project the response to immunotherapy. Alterations of certain molecules during tumor progression may also interfere with the interaction between immune cells or between immune cells and tumor cells, thereby disrupting the balance of immune resistance and activation in tumors [38]. However, how the genomic landscape in BRCA shapes and influences antitumor immunity is not yet clear.

Systematic analysis of tumor immune-related gene expression profiles can shed further light on the relationship between tumor genetics and TME. Genetic characterization may also assist in identifying suitable BRCA patients for immunotherapy. We clustered the cohort again based on DEGs between the previous ICI clusters, divided TCGA-BRCA patients into new ICI gene clusters, and defined ICI signature genes. Among these different ICI gene clusters, we discovered that ICI gene cluster C with the lowest levels of activated TILs, immune score, and stromal score exhibited an immune exhausted phenotype. On the contrary, ICI gene clusters A and B had higher inflammatory cell infiltration, immune scores, and stromal scores. And we also observed that ICI gene cluster B had a promising immune activation phenotype because of the highest content of macrophages, resting NK cells, memory activated CD4^+^ T cells, plasma cells, CD8^+^ T cells, etc. [60, 61]. Meanwhile, patients in ICI gene cluster B had the highest expression of PD-1 and PD-L1 and a more optimistic prognosis. We speculated that patients with ICI gene cluster B may be more likely to benefit from immunotherapy. The opposite is the case for patients with ICI gene cluster C, probably because their immune exhausted phenotype may lead to tumor cells evading the immune system and not responding to immunotherapy. Our study is following previous studies [62]. These findings suggested that combining the synthetic features of ICI profiles with expression patterns of immune-related genes in TME may become a promising approach to developing more precise immunotherapy regimens for BRCA patients.

Due to the high individual heterogeneity of TME, we used the ssGSEA method to establish ICI scores for patients in the TCGA-BRCA cohort and to quantify the ICI pattern for each patient. We found that the expression of most of the immune-related genes was higher in the group that had high ICI scores. GSEA analysis of the low ICI score group showed significantly enriched in TGF-*β* signaling pathway, epithelial mesenchymal transition, *etc.* Notably, TGF-*β* is a gene that is known to be involved in immunosuppressive pathways [37]. In addition, we found that our constructed ICI scores also correlated with TMB and neoantigens that also could predict response to immunotherapy [42, 63]. TMB levels and the number of neoantigens were significantly higher in the high ICI score group. Both survival analysis and stratified analysis indicated that higher ICI scores conferred a better prognosis for patients. Moreover, patients with high TMB and low ICI scores had the shortest survival. We also observed that different ICI score groups were associated with altered tumor driver genes (*e.g.*, *PI3KCA*, *TP53*, and *CDH1*) and gene copy number. High or low ICI scores also showed significant differences in sensitivity to certain other target drugs.

The ability of our established ICI score to predict response to immunotherapy in tumor patients was validated in a cohort of metastatic uroepithelial cancer patients treated with anti-PD-L1 agents (IMvigor210) [64]. Our results showed that ICI scores were significantly higher in patients who had a response to immunotherapy than in those who did not. Patients from the high ICI score group had longer survival and higher ORR. However, lacking data from a cohort of BRCA patients receiving immunotherapy, additional prospective trials are needed to validate these predictors that we constructed in the TCGA-BRCA cohort. In summary, our analysis has revealed environmental and genetic mechanisms affecting tumor-immune interactions in BRCA, and our constructed ICI score may serve as a powerful marker for predicting patient prognosis and the extent of benefit from immunotherapy.

## 5. Conclusions

In summary, our analysis has revealed environmental and genetic mechanisms affecting tumor-immune interactions in BRCA, and our constructed ICI score may serve as a powerful marker for predicting patient prognosis and the extent of benefit from immunotherapy.

## Data Availability

The datasets and original contributions used to support the findings of this study are included within the article and supplementary information files. Further inquiries can be directed to the corresponding author.

## Supplementary Materials

Supplementary 1 Supplementary Table 1: relative fractions of tumor-infiltrating immune cells of 1721 BRCA patients.

Supplementary 2 Supplementary Table 2: differentially expressed genes of ICI clusters in the TCGA-BRCA cohort.

Supplementary 3 Supplementary Table 3: ICI signature genes A and B.

Supplementary 4 Supplementary Table 4: clinical and survival information with ICI cluster and ICI gene cluster and ICI score of TCGA-BRCA patients.

Supplementary 5 Supplementary Table 5: gene set enrichment analysis (GSEA) of differentially expressed genes of ICI score groups.

Supplementary 6 Supplementary Table 6: drug estimated IC50 between high or low ICI score groups.
